# UV-B Induced Flavonoids Contribute to Reduced Biotrophic Disease Susceptibility in Lettuce Seedlings

**DOI:** 10.3389/fpls.2020.594681

**Published:** 2020-10-29

**Authors:** Emily R. McLay, Ana Clara Pontaroli, Jason J. Wargent

**Affiliations:** ^1^School of Agriculture and Environment, College of Sciences, Massey University, Palmerston North, New Zealand; ^2^BioLumic Limited, Palmerston North, New Zealand

**Keywords:** UV-B, flavonoids, *Bremia lactucae*, LED, photomorphogenesis, disease management, *Lactuca sativa*

## Abstract

Biotrophic disease is one of the largest causes of decreased yield in agriculture. While exposure to ultraviolet B (UV-B) light (280–320 nm) has been previously observed to reduce plant susceptibility to disease, there is still a paucity of information regarding underlying biological mechanisms. In addition, recent advances in UV-LED technology raise the prospect of UV light treatments in agriculture which are practical and efficient. Here, we characterized the capability of UV-B LED pre-treatments to reduce susceptibility of a range of lettuce (*Lactuca sativa*) cultivars to downy mildew disease caused by the obligate biotroph *Bremia lactucae*. Innate cultivar susceptibility level did not seem to influence the benefit of a UV-B induced disease reduction with similar reductions as a percentage of the control observed (54–62% decrease in conidia count) across all susceptible cultivars. UV-B-induced reductions to conidia counts were sufficient to significantly reduce the infectivity of the diseased plant. Secondary infections caused by UV-B pre-treated plants exhibited yet further (67%) reduced disease severity. UV-B-induced flavonoids may in part mediate this reduced disease severity phenotype, as *B. lactucae* conidia counts of lettuce plants negatively correlated with flavonoid levels in a UV-B-dependent manner (*r* = −0.81). Liquid chromatography–mass spectrometry (LC-MS) was used to identify metabolic features which contribute to this correlation and, of these, quercetin 3-O-(6”-O-malonyl)-b-D-glucoside had the strongest negative correlation with *B. lactucae* conidia count (*r* = −0.68). When quercetin 3-O-(6”-O-malonyl)-b-D-glucoside was directly infiltrated into lettuce leaves, with those leaves subsequently infected, the *B. lactucae* conidia count was reduced (25–39%) in two susceptible lettuce cultivars. We conclude that UV-B induced phenolics, in particular quercetin flavonoids, may act as phytoanticipins to limit the establishment of biotrophic pathogens thus delaying or reducing their sporulation as measured by conidia count. These findings highlight the opportunity for UV-B morphogenesis to be exploited through the application of UV-LED technology, as part of the development of next-generation, sustainable disease control tools.

## Introduction

Plant disease reduces the efficiency of crop production by decreasing potential yield by an estimated average of 16% (Oerke, 2006). Improved disease control techniques can help maximize crop production efficiency by reducing both losses of quality and yield. However, due to the versatility of plant pathogens, improvement of disease control can be an ambitious task. Methods which are highly effective such as chemical sprays and breeding for disease immunity often apply a heavy selection pressure resulting in the evolution of resistant pathogens (Strange and Scott, 2005). An integrated pest management system, which utilizes a combination of different control measures, can provide effect disease control while reducing the potential for development of pathogen resistance. It is therefore important to continue to develop and improve plant disease control tools for sustainable reduction of potential yield loss from disease.

The use of light-emitting diodes (LEDs) to provide a plant with specific light-signaling stimulation, such as ultraviolet B (UV-B; 280–320 nm), is an emerging approach which may be used to induce an increased defense against disease. UV-B is a short wavelength, high energy light that acts as a signal to induce a protective response in plants (Lee, 2016). Plants exposed to UV-B tend to become compacted, with thick leaves and high levels of polyphenols such as flavonoids (Jansen, 2002). UV-B can be recognized by the plant through a UV-B specific photoreceptor; UV-B resistance 8 (UVR8), or through less understood pathways (Jenkins, 2014). The high tolerance phenotype of UV-B-exposed plants may decrease susceptibility to biotic stress such as pathogens and insects (Wargent et al., 2005; Demkura and Ballare, 2012; Mewis et al., 2012; Schenke et al., 2019).

Previous studies reveal many overlaps between the response of a plant to UV-B light and pathogen attack. Ballaré et al. (2012) suggest UV-B acts in tandem with background light, in particular red: far red (R:Fr), to regulate partitioning of resources to vertical growth or defense. In low-competition scenarios, the high level of UV-B light (and R:Fr) acts as a signal to indicate upward growth is not required, and instead allocates resources to protective responses and branching (Mazza and Ballaré, 2015). However, the effect of UV-B exposure on the resistance of a plant to disease is a relatively unexplored area. The mechanism through which UV-B causes this increased resistance is also largely unknown. Demkura and Ballare (2012) conducted the most targeted investigation to date, in which the authors found a UV-B induced defense against *Botrytis cinerea* was UVR8 dependent and likely caused by increases to a syringyl type lignin. In addition to lignin, many phenolic compounds have also been observed to have antibacterial and antifungal activity (Rauha et al., 2000; Zhu et al., 2004; Gatto et al., 2011). A large number of these antimicrobial compounds—most commonly: querticin, and kaempferol flavonoids—are also upregulated by UV-B (Winter and Rostás, 2008; Hectors et al., 2012; Fraser et al., 2017). It is therefore hypothesized that UV-B induced flavonoids may have a role in a UV-B induced protection against disease.

We aimed to address this paucity of research using UV-B LED pre-treatments of seedlings as a potential disease control tool. Lettuce downy mildew caused by *Bremia lactucae* was chosen as a case study, due to past evidence toward a UV-B-induced resistance (Wargent et al., 2005) and the need for improved disease control methods in lettuce downy mildew. In addition, we hypothesized that UV-B pre-treatments can reduce disease severity in lettuce against *B. lactucae* and that UV-B-induced phenolics in part mediate this reduced disease severity phenotype.

## Materials and Methods

### Plant Material and Growth Conditions

Lettuce (*Lactuca sativa*) seeds were sown into seedling trays, with a cell size of 3 cm^2^, containing “Daltons Seedling Raising Mix” (Daltons^TM^, New Zealand). The tray was spread with a single layer of grade 3 medium vermiculite (Auspari Pty Ltd., Australia). Seedlings were grown for 14–17 days in a controlled temperature room (17°C) with a 10 h photoperiod providing 215 μmol m^–2^ s^–1^ from FL58W/965 super daylight deluxe fluorescent tubes (Sylvania Premium Extra, China). Capillary matting beneath the trays was watered daily until wet. Lettuce cultivars used comprised Casino, Pedrola (Terranova Seeds), El Dorado, Iceberg, Pavane, and Salinas (Richard Michelmore, UC Davis, CA, United States).

### UV-B Treatments

Light treatments were applied using a proprietary LED luminaire designed by BioLumic Ltd. (Palmerston North, New Zealand). Photosynthetically active radiation (PAR: 400–700 nm) light was supplied through red (630–690 nm) and blue (415–485 nm) light LEDs at a red:blue ratio of 0.8. The total PAR supplied was 215 μmol m^–2^ s^–1^. Our UV-B treatment was selected following an initial dose response study in a single lettuce cultivar (data not shown). UV-B treatment conditions consisted of either control (PAR only) or UV-B (PAR + 0.5 μmol m^–2^ s^–1^ UV-B), whereby the UV-B emission peaked at 300 nm. Light quality and quantity were confirmed with an OL756 double-scanning monochromator spectroradiometer (Optronic Laboratories, Orlando, FL, United States), and a portable spectrometer (Spectrilight ILT950, MA, United States) prior to each treatment. Light treatments were applied to seedlings at the 14 days after sowing stage, for a photoperiod of 10 h over 3 days in total. Following light treatment completion, plants received a darkness period of 14 h. Treatments were conducted in a 17°C controlled temperature room, with the LED luminaire acting as the sole light source. In summary, plants were propagated to 14 days post sowing under the white fluorescent lights, UV-B treatment was applied with the LED luminaire for 3 days, and then plants were returned to the white fluorescent lights for the infection and assessment period.

### Pathogen Inoculation and Disease Assessment

*Bremia lactucae* was isolated from a commercial lettuce production field (Otaki, New Zealand) during summer 2015. The *Bremia* isolate was identified as sextext code IBEB-C 36-01-00 or EU-B 16-63-40-00 using differential host set C as set by the International Bremia Evaluation Board (IBEB, 2013). Conidia were harvested by washing plants with sporulating tissue in sterile distilled water. The resulting conidia suspension was quantified using a hemocytometer (0.1 mL depth) (BRAND <^®^ counting chamber BLAUBRAND^®^ Neubauer improved, Merek, Auckland, New Zealand) and diluted to 10^5^ *B. lactucae* conidia mL^–1^ using sterile distilled water. Plants were misted with the conidia solution with a pressure sprayer until plants were saturated. Inoculated plants were kept in a misting tent at a temperature of 17°C and misted twice daily with water to encourage high humidity.

Conidia counts were taken 8, 11, or 12 days post-inoculation (DPI) depending on disease progression speed. Plants were washed in distilled water at a ratio of one plant per 20 mL. Leaves were washed in distilled water at a ratio of one leaf per 5 mL. An aliquot (5 μL) of the resulting suspension was pipetted into a hemocytometer (0.1 mL depth) (BRAND^®^ counting chamber BLAUBRAND^®^ Neubauer improved, Merek, Auckland, New Zealand) and conidia were counted in the four corner squares. The average of these four counts was multiplied by 10,000 to give a measurement in conidia mL^–1^. A pilot study did not show any UV-B mediated differences in leaf area following UV treatment (data not shown), therefore a focus was placed on conidia counts per plant/leaf. Each experiment was repeated three times. Four plants were washed together for each conidia count (number of conidia counts per light treatment = 13–16).

#### Secondary Infection Experiments

Secondary infection experiments used two sets of lettuce cv. Casino plants. Inoculum plants (“donor”) were treated with PAR + UV-B or PAR alone (control) as defined in Section “UV-B Treatments” and then inoculated with 10^5^ conidia mL^–1^ of *B. lactucae*. At 7 DPI, a second set of plants (recipient) were treated with PAR + UV-B or PAR alone (control), and were then placed into the misting tent containing donor plants. Inoculation of recipient plants by donor ones was encouraged by lightly pumping air over the plants followed by misting with water thrice a day for 7 days. Conidia counts of the recipient plants were taken at 11 DPI. Each experiment was repeated four times. Four plants were washed together for each conidia count (number of conidia counts per light treatment = 11).

### Quantitative Phenolics Measurements

Plants were measured for generalized flavonoid index using a Dualex leaf-clip instrument (Goulas et al., 2004) [Force A, Orsay; France] during the light treatment and disease period.

At 72 h, between treatment end and inoculation, sample plants were frozen in packets of three in liquid N_2_ and stored at −80°C. A modified version of the protocol used by Wargent et al. (2015) used to perform liquid chromatography–mass spectrometry (LC-MS). Frozen foliar material was crushed to a powder in liquid N_2_. Powdered leaf samples (150 mg each sample) were incubated overnight at 4°C in 1.5 mL of methanol/MQ/formic acid (80/20/1 v/v/v). Samples were diluted with methanol before analysis by LC-MS at Plant and Food Research, Palmerston North. LC-MS-grade methanol was obtained from Merek (Auckland, New Zealand). Ultrapure water (MQ) was obtained from a Milli-Q Synthesis system (Millipore, Billerica, MA, United States).

The LCHRMS system used was the same as that used in the previous study (Wargent et al., 2015), including a Dionex Ultimate^®^ 3000 Rapid Separation LC and a micrOTOF QII mass spectrometer (Bruker Daltonics, Bremen, Germany) fitted with an electrospray ion source. The LC contained an SRD-3400 solvent rack/degasser, an HPR-3400RS binary pump, a WPS-3000RS thermostated autosampler, and a TCC-3000RS thermostated column compartment. The column used was C68 (Luna Omega C18 100 × 2.1 mm id, 1.6 μm; Agilent, Melbourne, Australia) and it was maintained at 40°C. The flow rate was 0.400 mL min^–1^. Solvents were A = 0.2% formic acid and B = 100% acetonitrile, which set up a gradient over 20 min. The gradient was set up as 90% A, 10% B, 0–0.5 min; linear gradient to 60% A, 40% B, 0.5–9 min; linear gradient to 5% A, 95% B, 9–14 min; maintained at 5%A, 95%B, 14–18 min; linear gradient to 90%A, 10%B, 18–18.2 min; then returned to original conditions for next sample injection at 20 min. The injection volume was 1 mL. Mass spectrum (micrOTOF QII) parameters were as in Wargent et al. (2015). Each experiment was repeated three times, with all collected samples processed and run through LC-MS together (*n* = 9).

Analysis of raw output was completed by XCMS online (Gowda et al., 2014) to determine molecular features labeled with accurate mass and retention time. XCMS also grouped features into peak groups which likely represent a singular metabolite. Feature groups and representative mass were confirmed manually by curating spectral data using MZMINE (Pluskal et al., 2010). Intensities of features within a peak group were summed to determine feature area intensity. Identification (formula and compound name) of features was determined using MSDIAL and MSFINDER (Tsugawa et al., 2015; Lai et al., 2017). Proposed identification by MSFINDER was given a score of confidence and confirmed by comparison a composite sample MS/MS spectral data (MZMINE) to published spectrum and published literature.

### Phenolic Infiltration

Quercetin 3-O-(6”-malonyl-glucoside) (Q) was chosen from the list of candidate metabolites identified by LC-MS as having significant negative correlations with disease severity, as well as a strong certainty of identification. Compound standard of Q was ordered from Sigma–Aldrich (Sigma–Aldrich, Auckland, New Zealand). Published studies on polyphenol content were used to determine a control content of Iceberg/Crisphead type lettuce plant as 1.85 mg/100 g fresh weight Q (DuPont et al., 2000). Concentrations were adjusted for leaf weight, and infiltration volume of plants at 16 days old of each of the three cultivars and dilutions of the standards made to achieve a 1. 5-, 2. 5-, and 4-fold increase in each of El Dorado, Iceberg, and Salinas plants. These fold changes are based on UV-B-induced increases to levels of Q of 1.2–2.6-fold observed in LC-MS. The higher fold increase (x4) was selected to provide insight on how further increases to the compound could alter disease susceptibility.

Syringe infiltration is typically used in bacterial infiltration studies; however, it was adapted in this work for infiltration of phenolic compounds. Protocol loosely followed the leaf infiltration technique of Kim and Mackey (2008). Plants (16 days post sowing) were placed in a humid environment for 30 min to encourage stomatal opening. The oldest true leaf of each plant was marked for infiltration with permanent marker at the base of the leaf. Infiltrations were carried out by injections of either sterile distilled water (mock) or the Q solution using a 1 mL needle-less syringe into the back of the leaf at two points (one on each side of the midrib). Plants were infiltrated till the entire leaf had changed color indicating entry of the liquid (approximately 0.8 ± 0.1 mL). Plants were allowed to rest 17 h (5 h light, 12 h dark) prior to inoculation. Inoculation and disease assessment were carried out as described in Section “Pathogen Inoculation and Disease Assessment.” Each experiment was repeated four times. Each leaf was washed individually for each conidia count(number of conidia counts per light treatment = 14–52).

### Statistical Methods

R-3.6.0 (R Core Team, 2019) was used for statistical analysis, with base statistical package used unless otherwise specified. Graphs were produced using package ggplot2 (Wickham, 2016). Significant differences between means of normally distributed data such as conidia counts, feature area intensity, and dualex measurements were determined with *t*-tests or ANOVAs with Tukey *post hoc* (multcomp package; Hothorn et al., 2008). *P*-values of multiple comparisons were unadjusted. Relationships between multiple variables were investigated using linear models, two-way ANOVAs, principal component analysis, and Pearson correlation analysis.

## Results

### Lettuce Seedlings Exposed to UV-B Prior to Infection Release a Reduced Number of *B. lactucae* Conidia per Plant

**FIGURE 1:**
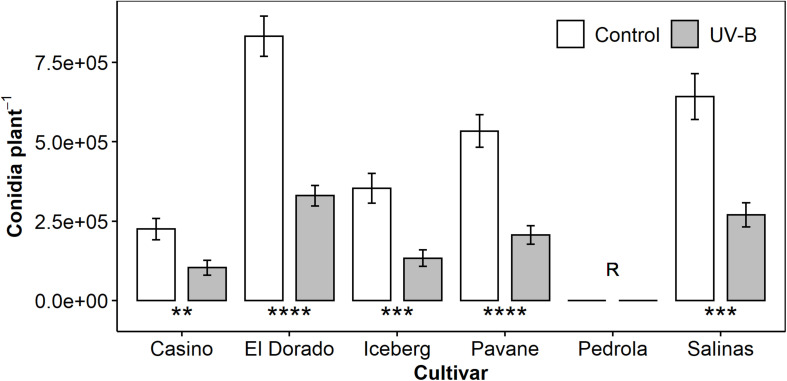
Mean *B. lactucae* conidia per plant was lower in UV-B (white bar)-pre-treated plants than control (gray bar) plants of multiple lettuce (*L. sativa*) cultivars (*n* = 13–16). Pedrola was completely resistant to *B. lactucae* infection as denoted by the letter R. Lettuce plants were treated with photosynthetically active radiation (PAR) + 0.5 μmol m^– 2^ s^– 1^ UV-B or PAR only (control) for 3 days and then inoculated with 10^5^ conidia mL^– 1^ of *B. lactucae*. At 12 days post-inoculation, plants were washed and the resulting conidia suspension counted. Error bars indicate 1 SE. Asterisks indicate significant differences between control and UV-B plants according to a *t*-test within each cultivar where **p < 0.005, ***p < 0.0005, ****p < 0.00005.

We assessed the effect of UV-B pre-treatment on the susceptibility of six lettuce cultivars to downy mildew disease using counts of *B. lactucae* conidia washed from infected plants at 12 DPI (*n* = 13–16). Cultivars ranged from highly susceptible (El Dorado) to fully resistant (Pedrola). UV-B pre-treatment reduced conidia harvested per plant at a similar level (54–62%) in all cultivars compared to plants exposed to PAR light only (Figure 1). Reductions in conidia count indicated a reduction in susceptibility to downy mildew disease.

### Secondary Infections Caused by UV-B-Pre-treated Plants Exhibit Enhanced Reduction of Disease Severity

As conidia are the primary inoculum of *B. lactucae* (Fall et al., 2016), UV-B induced reductions in conidia counts suggest a lower potential of secondary infections. To test this hypothesis, UV-B pre-treated or control lettuce plants were used as a source of *B. lactucae* inoculum (donor) to infect a new set of UV-B pre-treated or control plants (recipient). Treatments are described in the format of donor → recipient; e.g., UV→C indicates a UV-B pre-treated inoculum plant (donor) infected control plants (recipient). The disease symptoms of the secondary plant (recipient) were assessed (*n* = 11).

**FIGURE 2:**
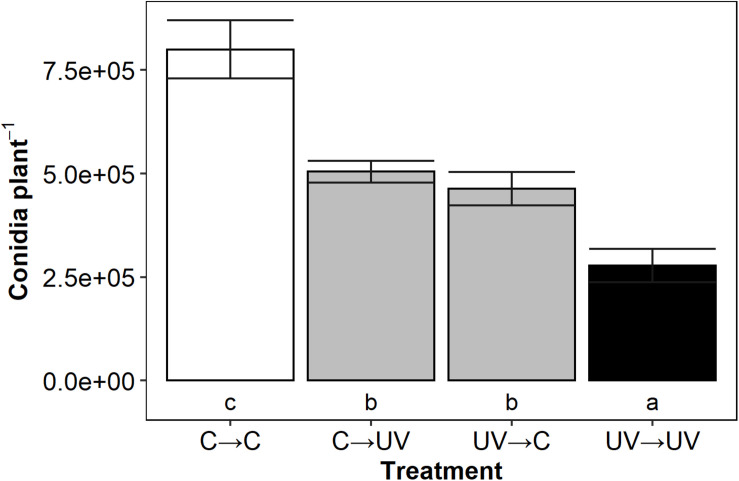
Mean *B. lactucae* conidia per lettuce (*L. sativa*) cv. Casino plant was lower in UV→UV plants than any other treatment combination (*n* = 11). Donor plants were treated with photosynthetically active radiation (PAR) + UV-B or PAR only (control) and then inoculated with 10^5^ conidia mL^– 1^ of *B. lactucae*. At 7 days post-inoculation, recipient UV-B or control treated plants were placed in a misting tent with donor plants. At 11 days after being placed in the misting tent, recipient plants were washed and the resulting conidia suspension counted. Treatments are coded in the format of donor→ recipient treatment where C = control and UV = UV-B-pre-treated. Lower case letters indicate significance groups (ANOVA Tukey HSD; *p* < 0.05). Error bars indicate 1 SE.

All secondary infections in which either the donor or the recipient or both plants received a UV-B pre-treatment had significantly lower conidia per plant than those that did not receive a UV-B pre-treatment (C→C) (Figure 2). Intermediate treatments reduced conidia count by 35% (C→UV, *p* < 0.0005) and 42% (UV→C, *p* < 0.0005) compared to C→C infections. When both donor and recipient plants received a UV-B pre-treatment (UV→UV), disease was further reduced (67%, *p* < 0.0005) compared to controls (C→C infections). Our results showed a progressive effect, where one set of UV-B plants (donor or recipient) caused an intermediate decrease in disease severity; however, when both sets of plants are UV treated (UV→UV), an amplified decrease occurred. This suggests that the amplified disease control effect is caused by the combination of a decreased inoculum from a UV-B pre-treated plant source and the additional UV-B protective response in the secondary plant.

### Reductions in Disease Severity Correlated With Increases in Overall UV-B Induced Flavonoids at Time of Inoculation

To determine whether UV-B induction of flavonoids contributed to an increase in disease protection, flavonoid levels were measured using a Dualex throughout the treatment and disease period and correlated with the resulting disease severity of infected plants. Regression analysis showed flavonoid level prior to inoculation negatively correlated with conidia count (24 h; *r* = −0.680, 48 h; *r* = −0.805, *p* < 0.05), however, was strongest at the time of inoculation (72 h) (*r* = −0.812, *p* < 0.05).

**FIGURE 3:**
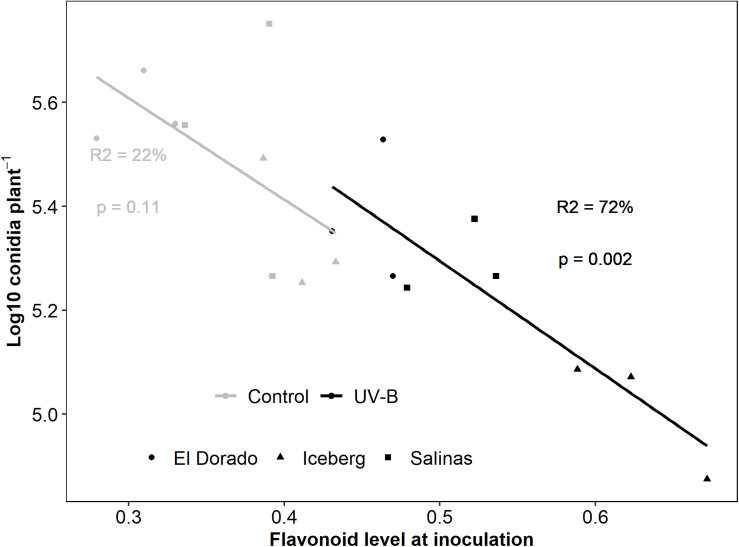
log10 Conidia count of *B. lactucae* decreases as flavonoid level of infected lettuce (*L. sativa*) plants increases. Lettuce plants were treated with photosynthetically active radiation (PAR) + 0.5 μmol m^– 2^ s^– 1^ UV-B or PAR only (control) for 3 days. Following treatment plants were non-destructively measured for flavonoids using a Dualex and then inoculated with 10^5^ conidia mL^– 1^ of *B. lactucae*. At 12 days post-inoculation, plants were washed in water and the resulting conidia suspension was counted using a hemocytometer (*n* = 9). UV-B-pre-treated plants (black points) drive this response with the regression (line) lost when control (gray points) are considered separately. Cultivars are indicated by point shape (El Dorado = circle, Iceberg = diamond, Salinas = square). Regression fit is indicated by the *R*^2^ values and model significance by *p*-value with text color indicating treatment (control = gray, UV-B = black)

UV-B-responsive flavonoids heavily drove this correlation (Figure 3). Separate models for UV-B-pre-treated and control plants indicated that although variation in flavonoids explained variation in conidia count in UV-B-pre-treated plants alone (*R*^2^ = 72%, *p* < 0.05), this was not the case for control plants alone (*R*^2^ = 22%, *p* = 0.11). To add, UV-B-pre-treated plants alone showed a very strong significant negative regression coefficient (*r* = −0.844) whereas control plants displayed a non-significant negative moderate regression coefficient (*r* = −0.491) between flavonoid level and conidia count. As the higher level of flavonoids in UV-B-treated plants can be attributed to UV-B response, we argue that UV-B-responsive flavonoids rather than general flavonoids contributed to the correlation and may have a role in reduction of disease susceptibility as demonstrated by conidia count.

### Untargeted Metabolomic Screening Revealed 36 Metabolomic Features Present in Lettuce Seedlings

Analysis of raw LC-MS data with XCMS (Gowda et al., 2014) revealed 1630 metabolic features (combinations of *m/z* and retention time). These formed 188 peak/feature groups. Using MZ mine (Pluskal et al., 2010) and MS-DIAL (Tsugawa et al., 2015), features with intensities over 1E3 were confirmed through comparison against the blank sample and examination of *m/z* patterns. The resulting 36 features (Supplementary Table 1) were then given putative identities using MS-FINDER or database searches to match precursor MS and resulting MS/MS spectrum using METLIN (Guijas et al., 2018) and MoNA (Horai et al., 2010). Multiple of the putative compounds we putatively identified have also been identified by spectral analysis of lettuce in previous studies (Supplementary Table 1). The majority of the putatively identified compounds were phenylpropanoids, especially flavonoids.

### UV-B Increased Level of Several Metabolomic Features

**FIGURE 4:**
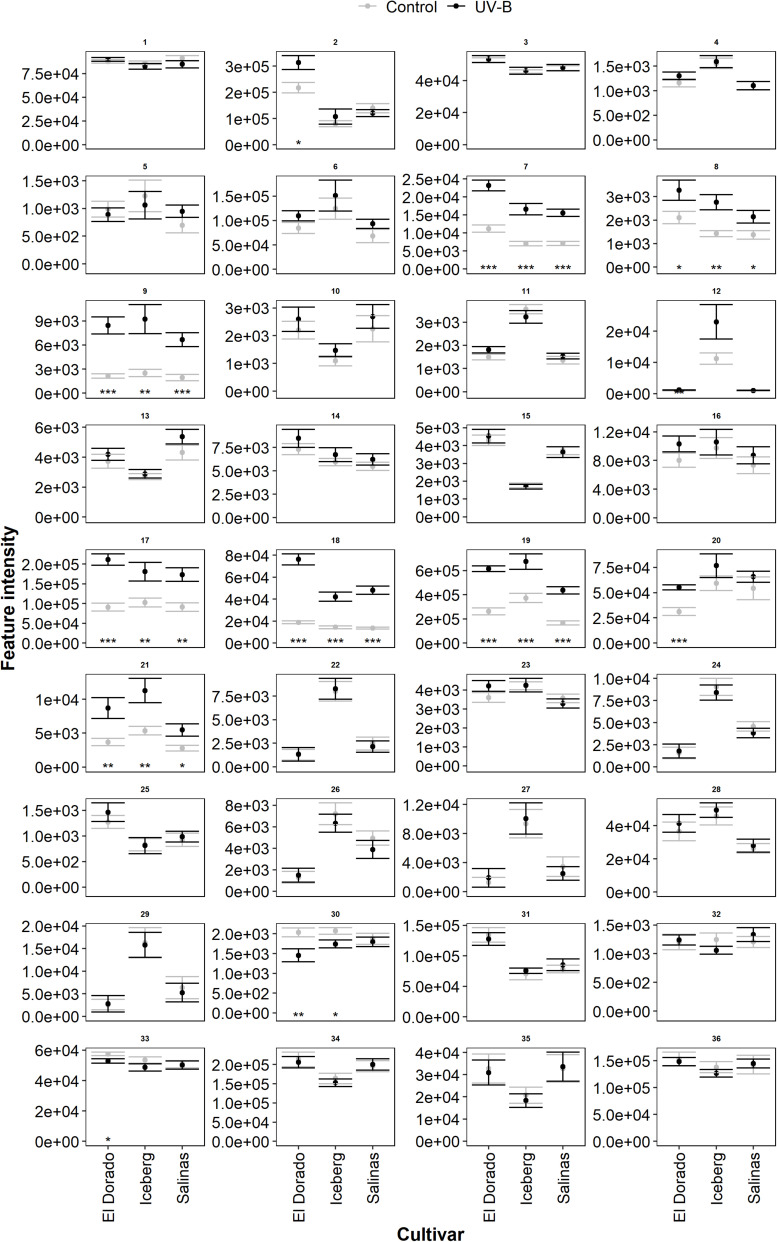
Mean intensity of all metabolomic features (Supplementary Table 1) in UV-B (black) and control (gray) plants of lettuce (*L. sativa*) cv.; El Dorado, Iceberg, and Salinas (*n* = 9). Plants were treated with photosynthetically active radiation (PAR)+UV-B or PAR only (control) for 3 days. Following treatment, samples were extracted for liquid chromatography–mass spectrometry (LC-MS) analysis. Significant differences between UV-B and control plants of each feature (panel) are indicated by asterisks (*t*-test, *p < 0.05, **p < 0.005, ***p < 0.0005). Error bars are 1 SE.

The metabolic features found in lettuce (cv. El Dorado, Iceberg, and Salinas) were expressed at different intensities (Figure 4, *n* = 9). The most intense features, indicating the highest abundance, were feature IDs 2, 6, 17, 19, 34, and 36. Although intensity indicated quantity, it does not necessarily indicate contributory importance of the corresponding metabolite to the disease response. Following UV-B treatment, many features experienced little or no change in feature intensity of UV-B-treated compared to control lettuce plants. Several features (7, 8, 9, 17, 18, 19, and 21) exhibited an overall higher feature intensity in UV-B-treated plants of each cultivar compared to control (*p* < 0.05). Features 2 and 20 were increased by UV-B exposure in cultivar El Dorado only (*p* < 0.05). Features which experienced a general increase by UV-B had a range of putative identities including a phenolic acid, flavonoid, and terpene. UV-B exposure decreased feature intensity across cultivars in very few cases. El Dorado and Iceberg UV-B-exposed plants had a reduced intensity of feature 30 (*p* = 0.0098 and 0.024, respectively), and UV-B-exposed El Dorado only had a reduced intensity of feature 33 (*p* = 0.027).

### Metabolomic Feature Intensity at the Time of Pathogen Inoculation Negatively Correlated With Conidia Count of Infected Lettuce Plants

A bivariate correlation analysis was run to determine relationships between disease severity and metabolomic feature intensity across all cultivars and treatments. Ten features showed a significant Pearson’s correlation (*r*) over 0.5 (positively or negatively) between conidia count and feature intensity (Table 1). All significant correlations (except feature ID 33) were negative indicating increases in feature intensity correlated with decreased disease severity. The feature intensity of feature 33, however, had a positive correlation with conidia count. The strongest negative correlations with conidia count were of features 11, 19, and 20.

**TABLE 1:** Significant correlations (*p* < 0.05) with a Pearson’s value over ±0.5 between feature intensity and conidia count across cultivars and treatments (*n* = 9).

Feature ID	Putative ID	Pearson correlation	Sig. (two-tailed)
9	Chlorogenic acid	−0.51	*
11	Unidentified	−0.62	*
17	Quercetin-3-glucuronide	−0.52	*
19	Quercetin 3-O (6-malonyl)-glucoside	−0.68	**
20	3,5-Dicaffeoylquinic acid	−0.61	*
22	Unidentified	−0.55	*
24	Unidentified	−0.56	*
27	Unidentified	−0.53	*
29	Unidentified	−0.52	*
33	Nitrogen containing lipid	0.55	*

Lettuce (*L. sativa*) cultivars (El Dorado, Iceberg, and Salinas) were treated with photosynthetically active radiation (PAR)+UV-B or PAR only (control) for 3 days. Following treatment, samples were taken for liquid chromatography–mass spectrometry (LC-MS), with the remaining plants inoculated with 10^5^ conidia mL^–1^ of *B. lactucae*. Plants were harvested for conidia count at 12 days post-inoculation. A bivariate correlation analysis was carried out on all LC-MS features and disease symptoms. Information on feature IDs is provided in Supplementary Table 1. Significance level is indicated by asterisks where *p < 0.05, **p < 0.005.

**FIGURE 5:**
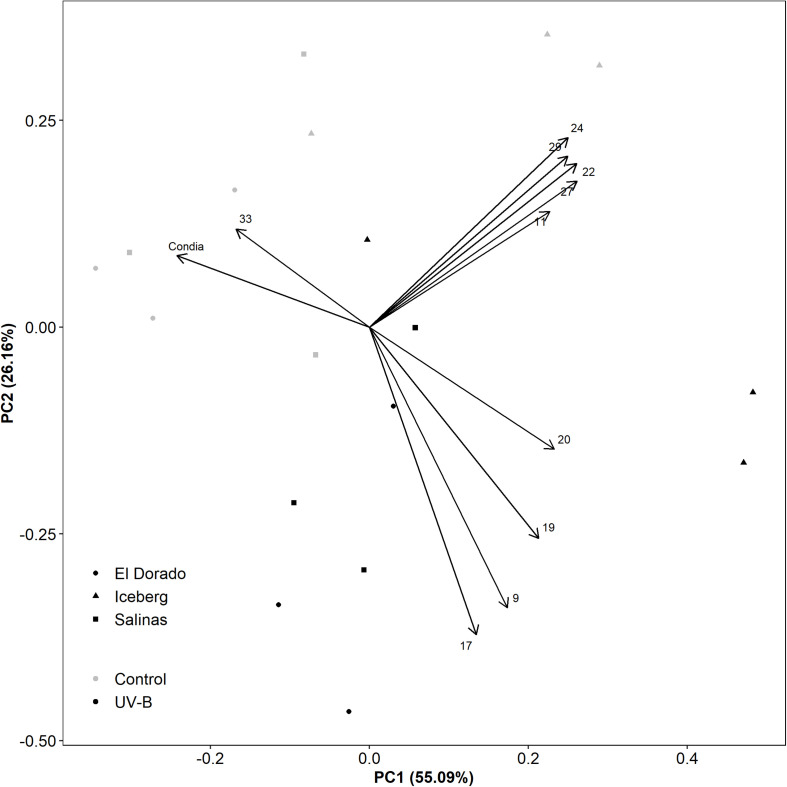
A principal component analysis evaluating disease assessment and key metabolite features of UV-B (black) and control (white) lettuce (*L. sativa*) plants of the cv. El Dorado (circle), Iceberg (diamond), and Salinas (square) (*n* = 9). Plants were treated with photosynthetically active radiation (PAR)+UV-B or PAR only (control) for 3 days. Following treatment, samples were taken for liquid chromatography–mass spectrometry (LC-MS) and the remaining plants were inoculated with 10^5^ conidia mL^– 1^ *B. lactucae*. At 12 DPI, plants were also harvested for conidia count.

The role of UV-B treatment and cultivar in correlations between conidia count and feature intensities was further investigated through a principal component analysis of key metabolic features and conidia count (Figure 5). UV-B and control plants were heavily separated diagonally across the first component (PC1) (55.09% of variation) and second component (PC2) (26.16% of variation). This indicated that both PC1 and PC2 can explain the majority (81.25%) of the treatment variation. Features 9, 17, 19, and 20 were separated from conidia count along both PC1 and PC2 suggesting they had a major influence on treatment effect.

The remaining features (11, 22, 24, 27, 29) formed a group high in both PC1 and PC2. These negatively correlated with conidia count along PC1 only. Although partially explained by treatment effect, this loading may also be influenced by cultivar effect. Cultivars El Dorado and Salinas are grouped together, however, heavily separated from Iceberg by PC1. While these features might be important for disease defense, correlations may also be driven by the low disease susceptibility of Iceberg.

### Direct Infiltrations of UV-B-Induced Phenolic Compounds Can Result in Decreased Disease Severity

**FIGURE 6:**
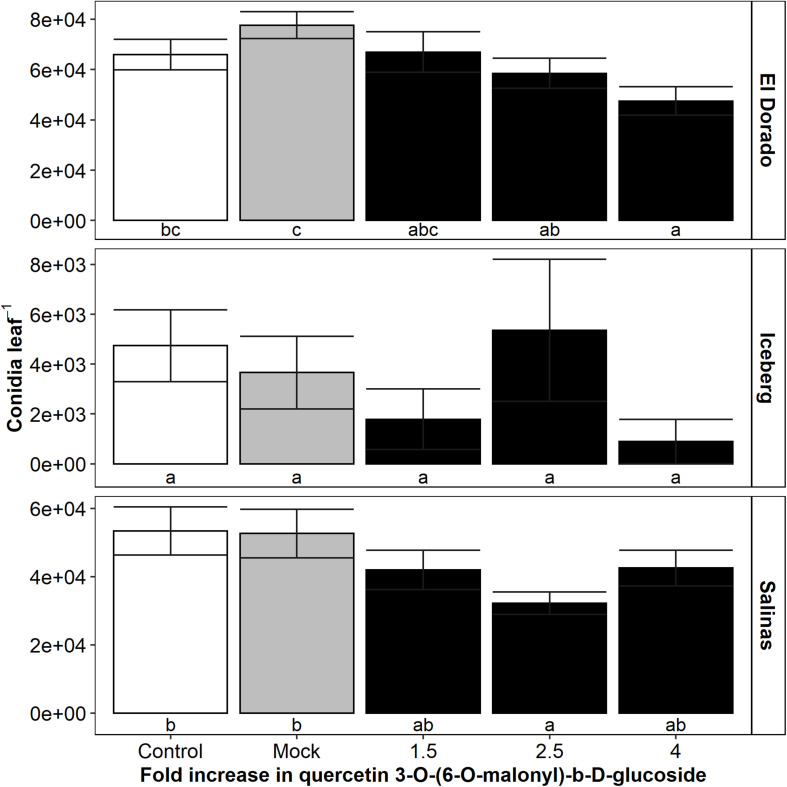
Mean *B. lactucae* conidia counts were reduced in lettuce (*L. sativa*) leaves infiltrated with a 2.5-fold increase in quercetin 3-O-(6-O-malonyl)-b-D-glucoside compared to mock infiltrated leaves in cultivars El Dorado and Salinas (*n* = 14–52). Lettuce plants were grown to 17 days old and then the oldest true leaf infiltrated with water (mock) or quercetin 3-O-(6-O-malonyl)-b-D-glucoside using a needleless syringe to reach a 1. 5-, 2. 5-, or 4-fold increase compared to a standard crisphead-type lettuce plant. Control plants were not infiltrated. Plants were then inoculated with 10^5^ conidia mL^– 1^ of *B. lactucae*. At 12 days post-inoculation, the infiltrated leaf was washed in water and the resulting conidia suspension counted. Error bars are 1 SE. Letters indicated significance groupings within each cultivar (ANOVA Tukey HSD; *p* < 0.05).

Feature 19, putatively identified as Quercetin 3-O-(6”-O-malonyl)-b-D-glucoside (Q), displayed the strongest negative correlation between intensity and conidia count. To determine if Q had a role in decreasing disease susceptibility, Q solutions were directly infiltrated into a leaf of each lettuce plant followed by inoculation with *B. lactucae* conidia. Infiltrated leaves were individually washed at 8 DPI, and the resulting conidia suspension counted (Figure 6) (*n* = 14–52).

Conidia counts in both El Dorado and Salinas leaves were decreased by addition of Q at 2.5-fold compared to mock infiltration (decrease of 25% in El Dorado; *p* = 0.029, 39% in Salinas; *p* = 0.024). Infiltration of El Dorado with fourfold Q also resulted in a significant reduction of leaf conidia compared to mock infiltration (39% decrease; *p* = 0.001). A non-significant trend suggests that El Dorado conidia count may decrease with increasing Q concentration. However, in Salinas, although leaf conidia count decreased more by an infiltration of 2.5 than 1.5-fold Q, conidia count was increased by fourfold Q infiltration. The peak for optimal Q concentration resulting in conidia decrease may be lower in Salinas than in El Dorado. Iceberg plants were unaffected by infiltration of Q. This is likely due to the low level of susceptibility of Iceberg plants as reflected by a high number (mock = 14% incidence) uninfected plants at 8 DPI compared to other cultivars (El Dorado = 98%, Salinas = 96% incidence in mock plants).

## Discussion

### UV-B Pre-treatment of Lettuce Plants Reduced Downy Mildew Disease Severity

#### UV-B Pre-treatment Has Potential to Reduce the Spread of Downy Mildew Disease

Ultraviolet B mediated reduction in *B. lactucae* conidia count of lettuce plants indicates UV-B pre-treated plants have a lower downy mildew disease susceptibility. Reduced conidia number also suggests a reduced inoculum, which in turn lowers potential secondary infections. The level of airborne conidia directly relates to the risk of downy mildew disease development and resulting yield loss (Fall et al., 2015). Lower conidia counts from UV-B-pre-treated plants as compared to untreated plants could be caused by several possibilities: (i) fewer conidiophores or conidia are produced in UV-B-pre-treated plants, (ii) attachment of conidia to conidiophores is increased in UV-B-pre-treated plants, or (iii) conidia are less stable and more likely to burst when produced on UV-B-pre-treated plants. Regardless of mechanism, our findings indicate that UV-B pre-treated plants exhibit interference with the development of conidiophores/conidia, and/or, UV-B-pre-treated plants have a lower potential risk of downy mildew infection spread, due to reduced levels of airborne *B. lactucae* conidia.

A reduced inoculum (conidia count) from infected UV-B pre-treated plants is sufficient to reduce disease symptoms of a secondary infected plant. When inoculum from a UV-B pre-treated donor plant was used to infect a control recipient plant (UV→C), a significantly lower disease severity was observed as compared with controls (C→C). Although in these experiments intermediate stages in which either the inoculum (donor) or the secondary infection (recipient) plants have experienced UV-B (C→UV or UV→C) displayed a moderate decrease in conidia count, the effect was not as great as when UV-B pre-treatment of both donor and recipient plants was applied. UV-B defense is therefore apparently accumulative, i.e., with greater cycle numbers of UV-B-treated plants that disease is spread through, the weaker disease severity becomes.

Our findings have implications for the agronomically beneficial effects of UV-B pre-treatment on disease spread dynamics in an applied setting. Although only two cycles of UV-B treatment were tested, it presents the possibility that severity of disease will decrease over infection cycles if they occur on UV-B pre-treated plants. Downy mildew disease is polycyclic in lettuce (Fall et al., 2016). Commonly, a small number of plants will be infected, develop symptoms, and become an inoculum source resulting in the infection of a secondary plant. This cycle continues until all nearby plants are infected unless disease control measures are taken. If infected plants were UV-B pre-treated, symptoms are limited, resulting in a reduced amount of inoculum on maturation of disease. Although it is likely secondary infections will still occur, disease may be restricted by UV-B-induced defenses resulting in a further reduced disease level in the recipient plant. The cycle continues with reduced inoculum levels as disease passes through UV-B pre-treated plants. Therefore, over cycles of disease produced on UV-B pre-treated plants, the *B. lactucae* inoculum is reduced, resulting in limitations in speed or area of spread of downy mildew disease.

#### UV-B Reductions in Conidia Count Are Comparable to the Efficacy of Current Commercial Control Tools

Ultraviolet B induced decreases of *B. lactucae* conidia count were comparable to current control measures of lettuce downy mildew. Cohen et al. (2008) showed that treatment of lettuce with several carboxylic acid amide (CAA) fungicides (mandipropamid, dimethomorph, and iprovalcarb) reduced *B. lactucae* conidia count of lettuce leaf disks by 48–55% when applied post infection. These reductions are slightly lower than the greatest reductions achieved by a UV-B pre-treatment in this work (58% reduction in conidia count). Conidia count reductions were, however, greater in leaf disks post infection treatment with benthiavalicarb (85% reduction) (Cohen et al., 2008) than they were following our UV-B-pre-treatments. These results show that UV-B can be nearly as successful at reducing downy mildew disease (as conidia count) as commercial standards, and with further development and as part of an integrated system, has a place as a disease control tool.

### UV-B Induced Flavonoids Contribute to Reductions in Disease Severity

Here, we have provided evidence toward a role of UV-B-induced flavonoids in an increased defense against downy mildew disease in lettuce. Reduction in disease severity (as conidia count) negatively correlated with UV-B-induced flavonoid level at the time of inoculation. LC-MS analysis of UV-B-induced metabolites indicates features which correlated with disease reduction included phenolic compounds such as phenolic acids and flavonoids. Infiltration of one such flavonoid [Quercetin 3-O-(6-O-malonyl)-b-D-glucoside] into lettuce leaves resulted in a decreased leaf conidia count in cultivars El Dorado and Salinas at levels slightly lower than that of UV-B pre-treatment. Therefore, UV-B-induced phenolics, in particular flavonoids, may be involved in mechanisms of UV-B induced reduction of disease severity; however, we cannot conclude they are responsible for the entire reduced susceptibility phenotype. A combination of UV-B morphogenic responses likely underlies the observed reduced conidia count, rather than the action of a single compound.

Quercetin and its derivatives have been associated with increased disease resistance in previous studies. However, a clear mode of action of quercetin in decreasing disease is not well established. Some studies suggest quercetin derivatives can have direct antimicrobial activity against bacterial and fungal pathogens (Rauha et al., 2000; Zhu et al., 2004; Gatto et al., 2011). Tao et al. (2010) suggests that a mechanism of antifungal activity could be through restricting initial spore germination and growth on the plant such as shown by quercetin-3-galactoside inhibition of germtube elongation of *B. cinerea*. Other studies suggest that quercetin has no direct anti-fungal action (Sanzani et al., 2008), but acts to enhance host resistance such as application of quercetin onto apples enhancing resistance to *P. expansum* (Sanzani et al., 2010). The glycosylated quercetin compound rutin has been shown to activate defenses against *Xanthomonas oryzae* pv. *oryzae*, *Ralstonia solanacearum*, and *Pseudomonas syringae* pv. *tomato* strain DC3000 in rice, tobacco, and *Arabidopsis*, respectively (Yang et al., 2016). This enhanced resistance could be due to quercetin acting as a pro-oxidant (Jia et al., 2010). Quercetin, although more commonly known as an anti-oxidant, can increase H_2_O_2_ levels resulting in the activation of defensive responses such as PR1 and PAL induction (Jia et al., 2010). Therefore, UV-B-induced upregulation of quercetin may have a direct antimicrobial effect on invading *B. lactucae* conidia or induce a host resistance response resulting in reduced conidia count.

#### Previous Studies Suggest UV-B-Related Flavonoids Are Suppressed by Biotic Stress

Schenke et al. (2011) summarized and explored the conflicting evidence that while many flavonoids have antimicrobial properties, flavonoids are also commonly downregulated by biotic stressors. Schenke et al. (2019) hypothesize that perhaps flavonoids may have had a role in protection against pathogens in evolutionarily early disease defense pathways, which later evolved into alternative, more successful phenylpropanoid derived defense pathways, such as lignin fortification. This would explain why flavonoids would be downregulated in order to funnel precursors into other branches of the phenylpropanoid pathway. Although as suggested by Schenke et al. (2019), UV-B induced flavonoids can be downregulated by biotic stress, this is not necessarily contradictory to our findings, as regardless of change post-infection, UV-B induced flavonoids present at the time of infection, i.e., induced prior to pathogen introduction, are the key to UV-B enhanced disease resistance in our work.

Host flavonoid response to disease appears to be very contextual with plant-disease system heavily influencing the accumulation of flavonoids. Observations in which flavonoids were downregulated in response to biotic stress commonly used elicitors alone (Lozoya et al., 1991; Logemann and Hahlbrock, 2002; Schenke et al., 2011; Serrano et al., 2012) often in a cell culture environment in which the success of the defense was not observed. Therefore, in these cases, although downregulation of flavonoids occurs, it is difficult to confirm if reduction in flavonoid production contributes toward an increased disease defense. In other cases, the regulation of flavonoids depends on the pathogen type. For example, confrontation of *Arabidopsis* with the obligate biotroph *Plasmodiophora brassicae* (Päsold et al., 2010) resulted in increases to flavonoids; however, a necrotophic interaction between onion and *Botrytis allii* resulted in flavonoid downregulation (McLusky et al., 1999). The regulation of (UV-B induced) flavonoids in disease defense may therefore not applicable as a broad statement, but rather to plant and pathogen types.

Changes in flavonoids as a response to disease may not always be an indicator of resistance, but as a susceptibility reaction. One method to alleviate some uncertainty about contribution of flavonoids to resistance is to examine the differential expression of flavonoid related genes during an incompatible (resistant host) versus an incompatible (susceptible host) plant–pathogen interaction. One such example was studied by Asai et al. (2014) in a model obligate biotrophic system comparable to that used in our work: *Arabidopsis* infected with pathogenic oomycete *Hyaloperonospora arabidopsidis* (causal agent of *Brassica* downy mildew). One of the major specialization steps in flavonoid synthesis is further differentiation of naringenin into dihydroflavonols. Dihydroflavonols include quercetins, such as Quercetin 3-O-(6”-O-malonyl)-b-D-glucoside found to have a high correlation with disease reduction in our work. An incompatible interaction, in which the plant is resistant to the pathogen, resulted in increased dihydroflavonol synthesis though upregulation of flavanone 3-hydroxylase (F3H) by 1.9-fold change at 1 day post-inoculation (Asai et al., 2014). However, this gene was heavily down regulated (−2.9-fold change) in a compatible interaction (Asai et al., 2014). Therefore, in an *Arabidopsis* downy mildew system, upregulation of F3H can act as a marker for resistance, providing evidence that flavonoids may still be upregulated as part of a successful disease defense. UV-B can also upregulate F3H (4.4-fold change; Ulm et al., 2004) strengthening evidence that UV-B can induce flavonoids that contribute to a biotrophic disease defense.

#### Disparities in the Involvement of Flavonoids in UV-B Induced Disease Defense May Be Due to Pathogen Lifestyle

Demkura and Ballare (2012) provided evidence against flavonoids contributing to UV-B-induced disease defense in a necrotrophic interaction. *Arabidopsis* mutants which had reduced function of chalcone synthase (*tt4-1*) and were therefore deficient in flavonoid synthesis maintained a UV-B-induced reduction to lesion size by the necrotrophic pathogen *B. cinerea*. In this case, UV-B-induced flavonoids were not required for UV-B-induced disease resistance. Schenke et al. (2019) provided mixed evidence toward a role of UV-B-induced flavonoids in necrotrophic disease defense. *Arabidopsis* mutants which lacked flavonoids (*chs/f3h*) had greater bacterial growth than wild type plants when infected with *Pseudomonas syringae* pv. *tomato* strain DC3000, indicating flavonoids have an antimicrobial activity; however, photomorphogenic UV-B pre-treatment failed to reduce *P. syringae* pv. *tomato* strain DC3000 growth rate (Schenke et al., 2019). Interestingly, UV-B pre-treatment of *Arabidopsis* plants did decrease the growth rate of *P. syringae hrcQ* mutants (Schenke et al., 2019). Paul et al. (2012) compared the use of UV transparent or opaque plastics to filter the level of UV from ambient sunlight reaching lettuce plants on the impact of both plant growth and biotic interactions (Paul et al., 2012). Exclusion of ambient UV reduced UV absorbing compounds (likely indicating flavonoids); however, this also resulted in decreased downy mildew infection, thus contradicting the disease relationship in our study. Paul et al. (2012) exposed/excluded both the pathogen and the plant from UV, thus the effect of UV on the pathogen itself will influence the resulting downy mildew infection.

The contrasting experimental evidence for flavonoids in a UV-B induced disease defense may be due to pathogen lifestyle. Necrotrophic pathogens tend to release toxic substances and enzymes to kill plant cells and extract nutrients from dead material (Glazebrook, 2005). On the contrary, biotrophic pathogens extract nutrients from living plant tissue; therefore, they must enter the plant less destructively, mostly occupying extracellular space (Hahn and Mendgen, 2001). Hence, biotrophic and necrotrophic pathogens have different invasion strategies and may be defeated by different plant defense responses. In response to biotrophic pathogens, less toxic antimicrobial compounds, such as flavonoids, might have a larger defensive role. To the best of our knowledge, our work is the first to dissect the role of UV-B-induced flavonoids in reducing biotrophic disease susceptibility.

#### UV-B Induced Flavonoid Phytoanticipins Are the Key to Increased Disease Protection

In our work, we identified that flavonoid level at the time of inoculation was key to increased disease resistance, i.e., the correlation between disease reduction and flavonoid level was highest at the time of inoculation. Therefore, UV-B-induced flavonoids with antimicrobial activity at time of infection (phytoanticipins) may reduce the ability of the pathogen to establish and reduce the severity of the resulting disease. Once the pathogen is recognized by the plant’s innate immune systems, downregulation of the flavonoid pathway may occur in some disease systems; however, this does not negate our argument for the importance of flavonoids which were induced by UV-B prior to infection. UV-B upregulation of phytoanticipins would satisfy both arguments that flavonoids contribute to defense against disease regardless of what occurs as part of an induced disease response.

## Conclusion

Ultraviolet B LED pre-treatments can reduce susceptibility to downy mildew disease caused by *B. lactucae* in lettuce (*L. sativa*). This reduction in disease severity is correlated with increases of phenolic compounds, in particular quercetin flavonoids. While some insights into mechanisms of induced defense are given, this remains a largely unexplored area. UV-B pre-treatments have significant potential to become a commercial tool for disease control, and further scientific investigation will advance the opportunity for commercial exploitation of such “clean green” sustainable disease control tools, as well as the development of UV-B pre-treatments for different crop-disease systems.

## Data Availability Statement

The datasets presented in this article are not readily available however raw metabolomic data files can be requested from BioLumic Limited. Requests to access the datasets should be directed to info@biolumic.com.

## Author Contributions

EM and JW planned and designed the research and analyzed and interpreted the data. EM performed the experiments and carried out metabolomics analyses. EM, AP, and JW co-wrote and approved the manuscript. All authors contributed to the article and approved the submitted version.

## Conflict of Interest

At the time of experimentation, JW was employed by Massey University and BioLumic Limited. At the time of manuscript submission, all authors were employed by BioLumic Limited, and JW was also employed by Massey University. The remaining authors declare that this study received partial funding from BioLumic Limited, and BioLumic Limited supplied the UV-B LED luminaires used in the study. BioLumic Limited was not involved itself in study design, collection, analysis, or interpretation of data.
