# Supplementary material for: UV-B Induced Flavonoids Contribute to Reduced Biotrophic Disease Susceptibility in Lettuce Seedlings
Source: Front Plant Sci. 2020 Oct 29;11:594681. doi: 10.3389/fpls.2020.594681 (PMC7673382; doi:10.3389/fpls.2020.594681)

Supplementary table 1. Putative identification of LC-MS (Liquid chromatography–mass spectrometry) peak groups. Lettuce (*L. sativa*) plants were treated with photosynthetically active radiation (PAR) + 0.5μmol m^-2^ s^-1^ UV-B or PAR only (control) for three days. Following treatment, samples were taken for LC-MS analysis with a negative polarity. Putative identification of metabolomic features are given, with their representative M-H mass charge (m/z) and retention time (RT) in minutes noted. If no evidence for M-H is found, then the highest intensity m/z is provided. Predicted formula and structure identity was determined using MS-DIAL and MS-FINDER. The corresponding error and score are given from these programs. In the case (**) where identity was determined through databases (Metlin and Mass Bank), no score is given. Two features (*) which strongly identify as chlorogenic acid were present. Identification of some features were confirmed by previous studies in lettuce. These are labelled as 1 : Ribas-Agustí et al. (2011), 2: Garcia et al. (2016), 3: García et al. (2017), 4: Sessa (2000), 5: Yang et al. (2018), 6: Ferreres et al. (1997) and 7: Romani et al. (2002).

**
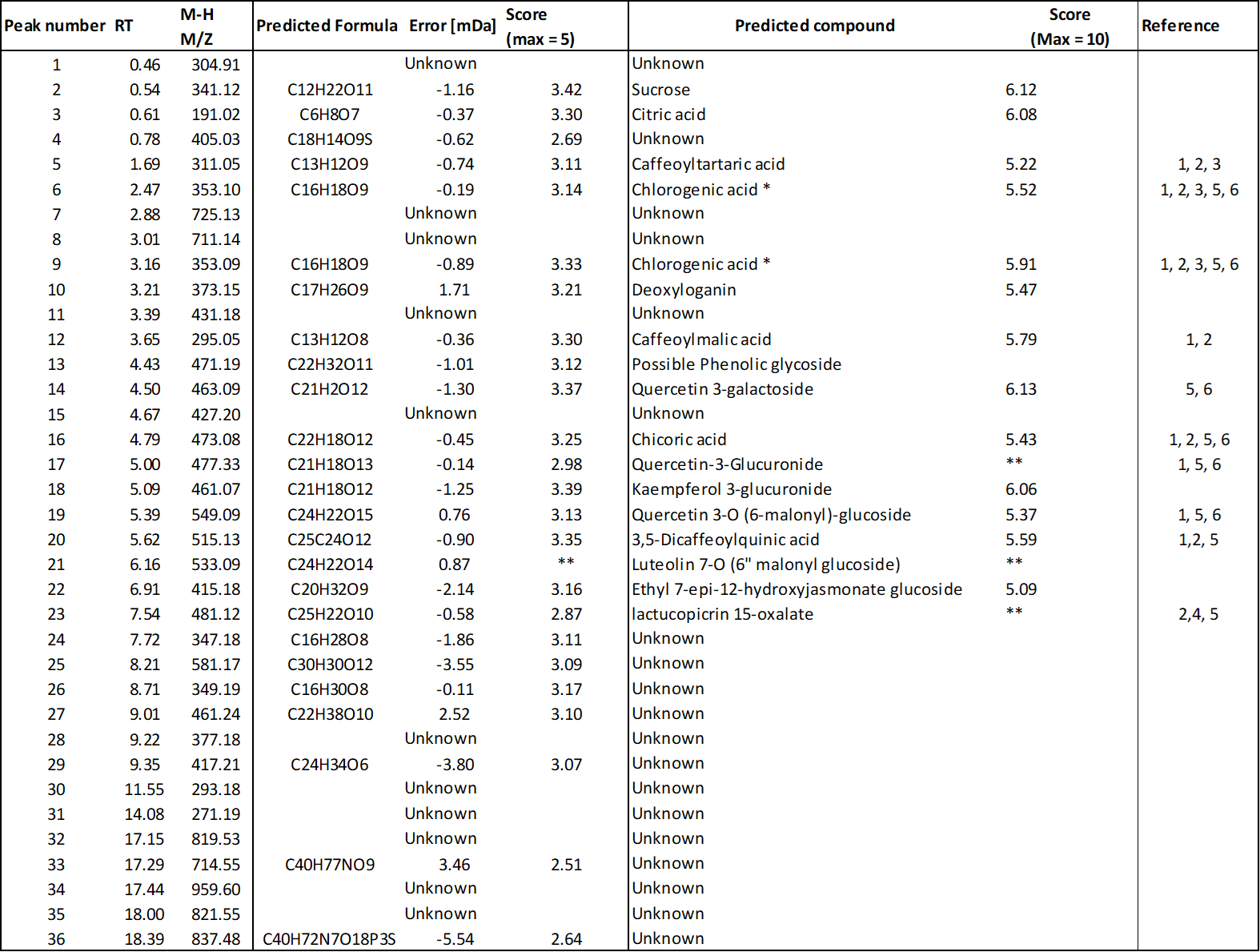
**


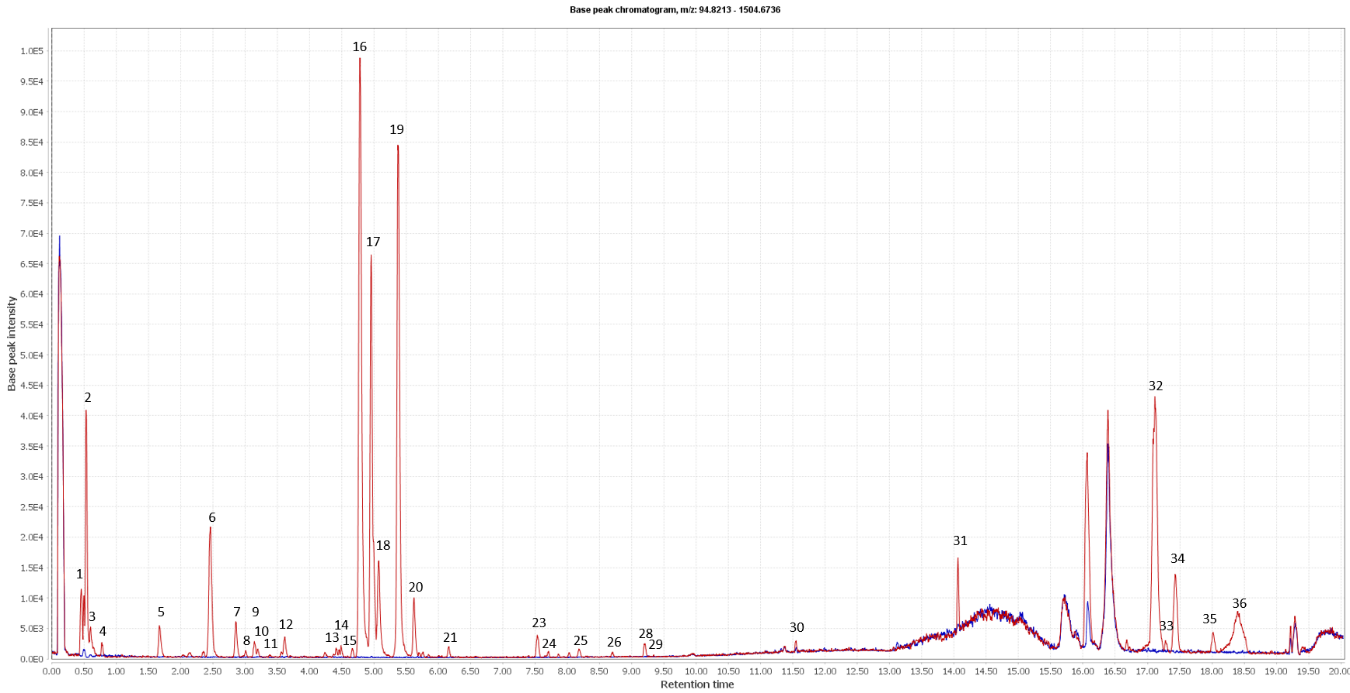

Supplement: Supplementary file 1 [file Table_1.DOCX]
